# Long-term CD4+ T-cell count evolution after switching from regimens including HIV nucleoside reverse transcriptase inhibitors (NRTI) plus protease inhibitors to regimens containing NRTI plus non-NRTI or only NRTI

**DOI:** 10.1186/1471-2334-11-23

**Published:** 2011-01-25

**Authors:** Carlo Torti, Antonella d'Arminio-Monforte, Anton L Pozniak, Giuseppe Lapadula, Giuliana Cologni, Andrea Antinori, Andrea De Luca, Cristina Mussini, Antonella Castagna, Paola Cicconi, Lorenzo Minoli, Andrea Costantini, Giampiero Carosi, Hua Liang, Bruno M Cesana

**Affiliations:** 1 University of Brescia, Italy; 2 S. Paolo Hospital, Milano, Italy; 3 Chelsea & Westminster Hospital, London, UK; 4 "S. Gerardo" Hospital, Monza, Italy; 5 Ospedali Riuniti, Bergamo, Italy; 6 INMI "L. Spallanzani" IRCCS, Rome, Italy; 7 Sacro Cuore Catholic University, Rome, Italy; 8 University of Modena and Reggio Emilia, Italy; 9 Vita-Salute University, Milan, Italy; 10 IRCCS Policlinico S. Matteo, Pavia, Italy; 11 University of Ancona, Italy; 12 Department of Biostatics and Computational Biology, University of Rochester Medical Center, Rochester, NY, USA; 13 Chair of Statistics, University of Brescia, Italy

## Abstract

**Background:**

Data regarding CD4+ recovery after switching from protease inhibitor (PI)-based regimens to regimens not containing PI are scarce.

**Methods:**

Subjects with virological success on first-PI-regimens who switched to NNRTI therapy (NNRTI group) or to nucleoside reverse transcriptase (NRTI)-only (NRTI group) were studied. The effect of the switch on the ongoing CD4+ trend was assessed by two-phase linear regression (TPLR), allowing us to evaluate whether a change in the CD4+ trend (hinge) occurred and the time of its occurrence. Furthermore, we described the evolution of the frequencies in CD4-count classes across four relevant time-points (baseline, before and immediately after the switch, and last visit). Finally, we explored whether the CD4+ counts evolved differently in patients who switched to NNRTI or NRTI-only regimens by considering: the overall CD4+ trends, the time to CD4+≥ 500/mm^3 ^after the switch, and the area-under-the-curve (AUC) of the CD4+ after the switch.

**Results:**

Eight hundred and ninety-six patients, followed for a median of 2,121 days, were included. At TPLR, hinges occurred in 581/844 (68.9%), but in only 40/581 (6.9%) within a time interval (180 days) compatible with a possible relationship to the switch; furthermore, in 19/40 cases, CD4+ counts appeared to decrease after the hinges. In comparison with the NNRTI group, the NRTI group showed CD4+ count greater at baseline (P = 0.0234) and before the switch (P ≤ 0.0001), superior CD4+ T-cell increases after HAART was started, lower probability of not achieving CD4+ ≥ 500/mm^3 ^(P = 0.0024), and, finally, no significant differences in the CD4+ T-cell AUC after the switch after adjusting for possible confounders (propensity score and pre-switch AUC). Persistence at CD4+ < 200/mm^3 ^was observed in 34/435 (7.5%) patients, and a decrease below this level was found in only 10/259 (3.9%) with baseline CD4+ ≥ 350/mm^3^.

**Conclusions:**

Switching from first-line PI to NNRTI- or NRTI-based regimens did not seem to impair CD4+ trend over long-term follow-up. Although the greater CD4+ increases in patients who switched to the NRTI-only regimen was due to higher CD4+ counts before the switch, several statistical analyses consistently showed that switching to this regimen did not damage the ongoing immune-reconstitution. Lastly, the observation that CD4+ T-cell counts remained low or decreased in the long term despite virological success merits further investigation.

## Background

The introduction of protease inhibitors (PI) as part of highly active antiretroviral therapy (HAART) in 1996 improved the clinical outcome of HIV-infected patients, decreased mortality rates by 3 to 10-fold and transformed HIV into a chronic disease [1]. The clinical benefits obtained by HAART (either for AIDS or non-AIDS related illnesses) were strongly correlated with CD4+ recovery [2,3].

Several studies demonstrated a positive effect of PI on CD4+ recovery over short-term follow-up [4-8], especially boosted by ritonavir [9,10]. The anti-apoptotic properties of PIs are thought to be the explanation for the better CD4+ responses [11]. Although other studies have failed to confirm this effect [12-15], it is important to assess whether simplification from PI-including to PI-sparing regimens may influence immune-recovery over long-term follow-up. Such studies are worth pursuing because treatment simplification is the most frequent reason for switching treatment in current clinical practice. Moreover, adverse events, intolerance and inconvenience of PI-containing regimens have led physicians to change an effective PI-based HAART to less complex and better-tolerated regimens [16].

Data on CD4+ evolution after switching PI have been limited by short follow-up and small numbers of patients [5,17-22]. Moreover, the patients were either experienced or naïve to antiretroviral drugs before HAART initiation [22], leading to conclusions not immediately applicable to a well defined population.

In planning a treatment simplification strategy, low cost and preservation of all the remaining classes (should HIV drug resistance eventually emerge) are two important benefits of switching to nucleoside reverse-transcriptase inhibitor (NRTI)-only regimens. In treatment simplification, the risk of virological failures, demonstrated with regimens containing only NRTI as first-line [23], has been limited to patients who have experienced previous virological failures and have accumulated HIV resistance to this class [17,18].

Therefore, the aim of the present study was to characterize the patterns of CD4+ responses in HIV-infected patients receiving virologically effective HAART regimens who changed from PI to regimens not containing PI: specifically, NNRTI or NRTI-only regimens. We evaluated whether this switch modified the ongoing evolutionary pattern of CD4+ T-cell count over long-term follow-up.

## Methods

### Design of the study

Retrospective inter-cohort study in patients achieving HIV-RNA < 500 copies/ml after 12 months of a first antiretroviral regimen including PI.

### Study patients

We followed 896 patients (median 2,121 days: IQR: 1,463-2,593) from MASTER, ICONA, Chelsea & Westminster, S. Raffaele and Modena HIV cohort databases, who initiated antiretroviral therapy with 2NRTI+PI (boosted or not by ritonavir) between 1997 to 2006. Follow-up was censored at loss to follow-up, death, two consecutive HIV-RNA≥ 500 copies/ml, or treatment discontinuation, whichever occurred first. The chosen threshold for viral suppression was HIV-RNA < 500 copies/ml since ultra-sensitive assays were not available throughout the study period.

Patients were grouped according to treatment change: 671 patients switched to non-NRTI (NNRTI)-based therapy (NNRTI group); 225 patients switched to NRTI-only (NRTI group).

The study was approved by the ethics committees of each participating center and the written consent was obtained from the participants.

### Influence of the switch on the ongoing trend of the CD4+ T-cell count

In order to provide reliable estimates, we evaluated patients with ≥ 10 CD4+ determinations. A two-phase linear regression [24] was fitted on the natural logarithm of the CD4+ count to assess the presence of a change in the ongoing CD4+ trend (hinge) and its possible relationship to the time of the switch. If a two-phase model did not fit better than simple linear regression (P > 0.10, conservatively), it was possible to conclude that no change in the CD4-trend had occurred; otherwise, we assessed whether the hinge occurred before, or ≤ 180 days after, the therapy switch (as a sensible time-lag for a cause-effect relationship), or later. The two-phase regression slope after the hinge, corresponding to at least 1 CD4+ T-cell/mm^3^/day decrease or increase, was considered to express a decreasing or an increasing trend, respectively.

### Overall trends of CD4+ T-cell count in patients who switched to NNRTI or NRTI-only regimens (unadjusted comparisons)

First, to compare CD4+ trends, we described the overall CD4-patterns across four relevant time-points (baseline, before the switch, immediately after and at the last visit) together with their percentage changes from baseline and the evolution of the frequencies in four CD4+ classes (≤ 200, 201-350, 351-500, and > 500/mm^3^). As a per-patient analysis, we recorded the frequencies of stable, decreasing or increasing patterns at the above four time-points, considering, for simplicity, the three classes: CD4+ ≤ 200, 201-350, and > 350/mm^3^.

Second, the Kaplan-Meier estimate of the cumulative probability of not obtaining CD4+ ≥ 500/mm^3 ^in at least two sequential determinations after HAART was calculated. The follow-up time of the patients with baseline CD4+ < 500/mm^3 ^was truncated at 1,460 days when 20% of the enrolled patients were still observed.

Third, a flexible nonparametric mixed-effects model (FNMEM) was applied taking individualization into account according to Liang [25]. A reference band of two standard errors width was calculated, centered at the average of the two estimated curves for the NNRTI and NRTI groups. If the two curves were not encapsulated in this reference band, they were considered, roughly, to be statistically different. Because the data were too sparse at the end of the treatment, data were cut at 2258 days when 30% of the enrolled patients were still under observation. Software R implementing the function LME [26], and Matlab^© ^[27] were used.

### Comparison of the CD4+ T-cell counts after the switch in patients who switched to NNRTI or NRTI-only regimens (adjusted comparisons)

We calculated the area under the curve (AUC) of the CD4-counts (log-transformed), adjusted for time (divided by the days of follow-up) as a summary statistic of the overall amount of CD4+ at each time point. In particular, we derived: (i) the pre-switch AUC from before the start of therapy (median 14 days, interquartile range, IQR: 0-36 days) to the therapy switch, and (ii) the post-switch AUC from the first CD4-count immediately after the switch (median 95 days, IQR: 63-126 days) until the last observation.

To compare non-randomized groups, the propensity score of belonging to the NNRTI or NRTI group was calculated using a multiple logistic model [28]. The effect of the switch on the post-switch AUC was tested by ANCOVA (NNRTI group vs. NRTI group) with, as covariates, the propensity score and the pre-switch AUC to adjust for differences in the CD4+ T-cell count before the switch or by the Wilcoxon rank-sum test for unadjusted comparisons. Qualitative variables were compared with a χ2 test.

Owing to the large sample size of at least 225 patients, it was possible to demonstrate a very low effect size of about 0.25 (a difference of 25% in the variability of the phenomenon investigated), which could be clinically insignificant.

Statistical analyses were performed using SAS^© ^version 9.1.

## Results

### Patients' characteristics at baseline

Regarding the baseline characteristics of the 896 patients (Table 1), no statistically significant differences were found between the two groups except for calendar year at study initiation and CD4+ counts.

**Table 1:** Characteristics of patients at baseline

Qualitative variable - N (%)	NNRTI group N = 671	NRTI group N = 225	P
Gender [Male]	536 (79.9%)	182 (80.9%)	0.7429
Risk factor for HIV acquisition			0.6749
IVDU	148 (22.1%)	46 (20.4%)	
Homo-bisexual	198 (29.5%)	65 (28.4%)	
Heterosexual	254 (37.9%)	83 (36.9%)	
Other	16 (2.4%)	9 (4%)	
Unknown	55 (8.2%)	22 (9.8%)	
Nationality			0.6511
Europe or North America	608 (90.6%)	208 (92.4%)	
Other	41	12	
Unknown	22	5	
HCV-Ab			0.8498
Positive	169 (25.2%)	55 (24.5%)	
Negative	432 (64.4%)	149 (66.2%)	
Unknown	70 (10.4%)	21 (9.3%)	
CDC '93 Class C	200 (29.8%)	64 (28.4%)	0.6982
CD4+ classes			0.0782
≥ 200/mm^3^	342 (51.0%)	93 (41.3%)	
201-350/mm^3^	146 (21.8%)	56 (24.9%)	
351-500/mm^3^	101 (15.0%)	45 (20.0%)	
> 500/mm^3^	82 (12.2%)	31 (13.8%)	
Centre			0.1448
Master	399 (59.5%)	115 (51.1%)	
Chelsea & Westminster	97 (14.5%)	38 (16.9%)	
ICONA	139 (20.7%)	60 (26.7%)	
HSR+Modena	35 (5.3%)	12 (5.3%)	
Calendar year at study initiation			0.0137
1997-2000	264 (39.3%)	70 (31.1%)	
2001-2003	309 (46.1%)	129 (57.3%)	
2004-2006	98 (14.6%)	26 (11.6%)	
Boosted-PI as first line	78 (11.6%)	30 (13.3%)	0.4957
**Quantitative variables**			
Mean (SD) age [years]	39 (35-45)	38 (34-44)	0.3680
Mean (SD) pre-treatment HIV-RNA [log_10 _copies/ml]	5.012 (4.415-5.430)	5.000 (4.511-5.519)	0.6269
Median (IQR) CD4-count [cells/mm^3^]	196 (86-377)	249 (103-403)	0.0234

In fact, more patients in the NRTI group initiated HAART in the period 2001-2003 than patients in the NNRTI group. Moreover, there was a statistically significant difference between the CD4+ counts in favour of the NRTI group (P = 0.0234) at baseline. This difference was also apparent for the CD4+ classes, though at a borderline significance level (P = 0.0782). Also, the CD4+ AUC before the switch was significantly greater (P < 0.0001) in the NRTI group (mean ± standard deviation, SD: 499 ± 263.9 cells/mm^3 ^per day) than in the NNRTI group (418 ± 236.6 cells/mm^3 ^per day).

Regarding specific drugs that could influence CD4+ count [29,30], zidovudine was prescribed in 501/896 (55.92%) patients, tenofovir in only 8 patients, while the association of tenofovir/didanosine was never prescribed. Boosted PI as first line regimens were prescribed in 108/896 (12.05%) patients. No statistical differences in the prescription of these drugs were found between the two groups.

### Influence of the switch on the ongoing trend of the CD4+ T-cell count

Patients in the group 1 were switched to regimens containing either efavirenz (373/671 = 55.59%) or nevirapine (298/671 = 44.41%). Zidovudine was prescribed in 350/671 (52.16%) patients, tenofovir in 44/671 (6.56%) and the association of tenofovir/didanosine in 15/671 (2.23%). Regarding the group 2, zidovudine was prescribed in 186/225 (82.67%) patients (P < 0.0001 vs. group 1), tenofovir in 18/225 (8%) and the association of tenofovir/didanosine in only 2 patients.

Among 844/896 (94.2%) patients with ≥ 10 CD4+ determinations, a two-phase linear regression model fitted better (P < 0.10) than a simple linear regression in 436/627 (69.5%) patients in the NNRTI group and in 145/217 (66.8%) in the NRTI group. The hinges occurred at medians of 617 days (IQR: 336-1041) and 737 days (IQR: 392-1223) before the therapy switch and at medians of 308 days (IQR: 135-579) and 404 days (IQR: 218-722) after HAART initiation in the NNRTI and in the NRTI group, respectively.

Table 2 shows the percentages of patients with increasing, stationary or decreasing trends after the hinge, stratified by the time of its relationship with the switch: before, ≤ 180 days or >180 days. Estimates of the trends are also shown. Overall in the two groups, the hinges occurred within 180 days after the switches in only 40/581 (6.9%) patients, and the CD4-counts appeared to decrease after the hinges in only 19/40 (47.5%). Thus, the switches did not appear to be related to decreasing CD4+ trends in 93.1% of the cases. In addition, decreasing CD4+ trends occurred in 122 (22.5%) patients out of the 541 with hinges before (N = 65) or more than 180 days after (N = 57) the switch.

**Table 2:** Characteristics of the hinges in the trends of CD4+ T-cell counts (two-phase linear regression model)

NNRTI group N = 436/627 (69.4%)			NRTI group N = 145/217 (66.8%)		
**Time of the hinge relative to the switch (categories)**					

Before the switch	≤ 180 days from the switch	>180 days from the switch	Before the switch	≤ 180 days from the switch	>180 days from the switch
N = 325/436 (74.5%)	N = 31/436 (7.1%)	N = 80/436 (18.3%)	N = 118/145 (81.4%)	N = 9/145 (6.2%)	N = 18/145 (12.4%)

**Increasing CD4+ trend after the hinge relative to the pre-switch trend (categories)**					

N = 210/325 (64.6%)	N = 7/31 (22.6%)	N = 18/80 (18.7%)	N = 68/145 (46.9%)	N = 3/9 (33.3%)	N = 1/18 (5.5%)

**Estimates of the increasing trend (median CD4+/mm**^**3**^/day, IQR or min-max*/individual^**$ **^**values where indicated)**					

1.0003	1.0003	1.0005	1.0003	*min-max:	
1.0002 to 1.0005	1.0002 to 1.0082	1.0001 to 1.0015	1.0002 to 1.0004	1.0001 to 1.00025	^$^1.0001

**Stationary CD4+ trend after the hinge relative to the pre-switch trend (categories)**					

N = 68/325 (20.9%)	N = 8/31 (25.8%)	N = 20/80 (25%)	N = 32/145 (22.1%)	N = 3/9 (33.3%)	N = 5/18 (27.8%)

**Decreasing CD4+ trend after the hinge relative to the pre-switch trend (categories)**					

N = 47/325 (14.5%)	N = 16/31 (51.6%)	N = 45/80 (56.2%)	N = 18/145 (12.4%)	N = 3/9 (33.3%)	
					N = 12/18 (66.7%)

**Estimates of the decreasing trend (median CD4+/mm**^**3**^/day, IQR or min-max* values where indicated)					
-1.0002	-0.99981	-1.0007	-1.0003	*min-max:	-1.0004
-1.0001 to -1.0004	-0.99959 to -0.999945	-1.0029 to 1.0003	-1.0005 to -1.0002	-0.99585 to -0.99988	-1.0015 to -1.0003

### Overall trends of CD4+ T-cell count in patients who switched to NNRTI or NRTI-only regimens (unadjusted comparisons)

Medians, percentage increases and strata of CD4-count during the study are shown in Table 3. Estimates were based on medians of 21 (IQR: 14-27) and 21 (IQR: 15-27) CD4+ determinations per patient in the NNRTI and in the NRTI group, respectively. CD4-counts increased by at least double the baseline, especially from baseline to the period immediately after the switch, while smaller increases occurred afterwards. Also, the CD4+ AUC before the switch was significantly greater (P < 0.0001) in the NRTI group (mean ± SD: 499 ± 263.9 cells/mm^3 ^per day) than in the NNRTI group (418 ± 236.6 cells/mm^3 ^per day), as well as the CD4+ count before the switch (P < 0.0001).

**Table 3:** CD4+ T-cell counts at relevant time points of the study

	**Pre-switch**		**Post-switch**		**Last observation**	
	**NNRTI** **group**	**NRTI** **group**	**NNRTI** **group**	**NRTI** **group**	**NNRTI** **group**	**NRTI** **group**
Median (IQR): days from HAART	788 (489-1200)	1002 (646-1432)	896 (595-1331)	1103 (759-1620)	2080 (1398-2567)	2220 (1638-2663)
Median (IQR) CD4-count [cells/mm^3^]*	468 (321-660)	561 (402-758)	478 (322-683)	568 (386-799)	538 (389-742)	634 (446-871)
CD4+ increase (%) from baseline	138.8	125.3	143.9	128.1	174.5	154.6
N (%) CD4+ classes^$^						
<200/mm^3^	61 (9.1%)	10 (4.5%)	44 (6.6%)	12 (5.3%)	30 (4.5%)	10 (4.4%)
201-350/mm^3^	140 (20.9%)	36 (16.0%)	147 (21.9%)	33 (14.7%)	92 (13.7%)	26 (11.6%)
351-500/mm^3^	167 (24.9%)	48 (21.3)	165 (24.6%)	45 (20.0%)	168 (25.0%)	33 (14.7%)
>500/mm^3^	303 (45.1%)	131 (58.2%)	315 (46.9%)	135 (60.0%)	381 (56.8%)	156 (69.3%)

*Comparisons at Pre-switch: P < 0.0001; Post-switch: < 0.0001; Last observation: < 0.0001;

^$^Comparisons at Pre-switch: P < 0.0038; Post-switch: < 0.0073; Last observation: < 0.0044;

Of note, the percentage of patients with CD4+ ≤ 200/mm^3 ^was always about 5%. Among patients with baseline CD4-count ≤ 200/mm^3^, a transition to upper CD4+ strata occurred in 316/342 (92.4%) and 85/93 (91.3%) in the NNRTI and in the NRTI group, respectively. Among patients with baseline CD4+ count between 201 and 350/mm^3^, 126/146 (86.3%) in the NNRTI group and 53/56 (94.64%) in the NRTI group had an increase to upper CD4+ strata, while two (1.37%) in the NNRTI group and two (3.57%) in the NRTI group had a decrease to ≤ 200 CD4+/mm^3^. Lastly, a decrease to CD4+ T-cell count ≤ 350/mm^3 ^was found in 7/183 (3.8%) and 3/76 (3.95%) patients with baseline CD4+ T-cell count > 350/mm^3 ^in the NNRTI group and in the NRTI group, respectively.

**Figure 1:**
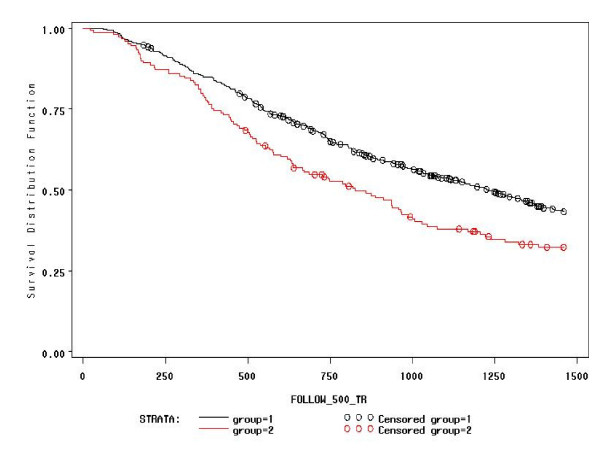
**Proportion of patients not having CD4+ ≥ 500/mm^3 ^at each time point (the NNRTI group is represented with the black line and the NRTI group is represented with the red line)**. Legend to figure 1: Time (days) is represented on the x-axis, truncated at 1,460 days when 20% of the study patients were still observed.

Survival probabilities for not obtaining CD4+ ≥ 500/mm^3 ^in at least two sequential determinations after HAART are depicted in Figure 1. In 637 patients with baseline CD4+ < 500/mm^3 ^followed for a median of 943 (IQR: 505-1619, but truncated to 1460) days, the probability of not achieving a confirmed CD4-count ≥ 500/mm^3 ^was higher in the NNRTI group than in the NRTI group (Log-Rank = 9.2309; df = 1; P: 0.0024). In particular, at one year it was 0.85 (95%CI = 0.81-0.89) vs. 0.79 (0.73-0.85), and at three years 0.54 (0.50-0.58) vs. 0.38 (0.30-0.46).

**Figure 2:**
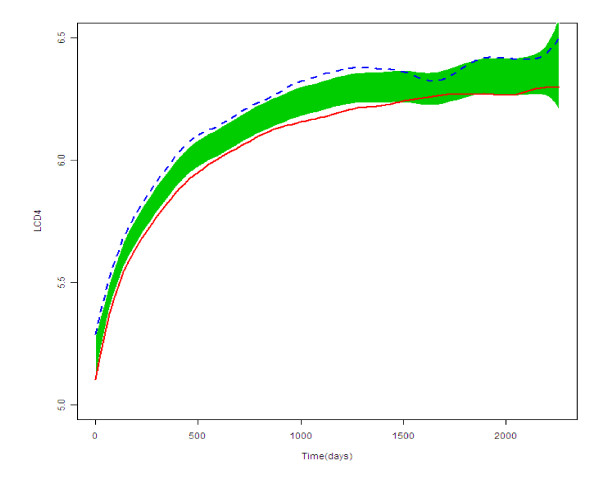
**Population curves for group features and the reference band (shaded area) for the NNRTI group (represented with the continuous red line) and for the NRTI group (represented with the dotted blue line)**. Legend to figure 2: The figure represents the scatter plot of the logarithm of the CD4+ count (LCD4, y-axis) against time for the two groups under study.

Regarding FNMEM, the two population curves of the CD4+ increase after the commencement of HAART to the last observation for the NNRTI group and the NRTI group and the reference bands [25] are depicted in Figure 2. The CD4+ increase appeared to be greater in the NRTI group than in the NNRTI group.

### Comparison of the CD4+ T-cell counts after the switch in patients who switched to NNRTI or NRTI-only regimens (adjusted comparisons)

There was a statistically significant difference in the CD4+ counts (Table 3) in favour of the NRTI group immediately after the switch and at the last observation (P < 0.0001); this difference was also consistently observed for the CD4+ classes (P = 0.0073, and P = 0.0044, respectively).

Furthermore, the CD4+ AUC was significantly greater (P < 0.0001) in the NRTI group (mean ± SD: 661 ± 300.0 cells/mm^3 ^per day) than in the NNRTI group (574 ± 266.5 cells/mm^3 ^per day). However, the change in the CD4+ AUC (difference between before and after the switch) proved statistically insignificant (P = 0.0586: NNRTI group increase of 155.6 ± 132.8 cells/mm^3 ^per day; and NRTI group increase of 162.3 ± 134.7 cells/mm^3 ^per day).

## Discussion

We followed a cohort of antiretroviral-naïve patients initiating HAART containing PI over a long term-follow-up of 2,121 days (median) under virologically effective regimens, switching to regimens not including PI. Linear regression analysis seemed to rule out the possibility that switching from PI to alternative regimens caused immune damage. In fact, in 31.2% of patients, no hinges could be detected, while among those who presented a hinge, its relationship to the switch was reliable (≤ 180 days) in only 6.9% (40/581) patients. Moreover, a decreasing trend was demonstrated in only half (19/40). While the CD4+ T-cell count increased more in patients who were switched to NRTI-only regimens than in those who were switched to NRTI+NNRTI, this was probably because, in the NRTI group, the CD4+ T-cell count at baseline was higher and CD4+ T-cell count increase before the switch was greater. In fact, ANCOVA models adjusted both for the propensity score and for pre-switch AUC demonstrated no significant differences in the post-switch AUC.

Regarding the main object of this paper (the evaluation of the influence of the switch on CD4+ trend) our results are consistent with the meta-analysis by Bucher et al. [31] on simplification studies over a maximum follow-up of 52 weeks and with that of Le Moing et al. [22] who conducted an observational study over a median follow-up of 57 months, demonstrating a non-detrimental effect of switching from PI to NNRTI (229 patients) or NRTI-only (34 patients). However, our study is based on many more patients (896) who switched, and over a longer follow-up (median of about 72 months). More recently, Elzi et al. [32] demonstrated that switch "per se" was not associated with a increased risk of virological failure. Moreover, no difference in the median CD4+ count increase at 12 months was noted among switchers and individuals who did not change their treatment [32]. The present study adds data regarding CD4+ increase over a longer follow-up, and about a certain class regimen: the fact that the comparison of CD4+ changes between the two groups from before to after the switch proved statistically insignificant allows us to conclude that the ongoing increase of CD4+ T-cells was not significantly influenced by the switch to the first (NNRTI) or to the second (NRTI) regimen under study.

Other studies assessed CD4+ increases upon initiation of antiretroviral therapy indicating that CD4+ increases were better with PI (especially boosted by ritonavir) compared with NNRTI or triple NRTI regimens even though virological responses were similar among the three regimens or better with NNRTI than with PI boosted by ritonavir [4-10]. Our analysis performed in patients who already attained undetectable HIV-RNA and maintained it for quite a long time did not suggest that abandoning PI was able to influence the ongoing trend in CD4+ count. It could be that the protective effect of PIs (probably mediated by anti-apoptotic mechanisms [11]) is more important when the immune system is more hyper-activated or damaged for the presence of actively replicating HIV (as it happens in antiretroviral naïve patients off treatment) than when the immune system has already been restored (at least in part) through the HIV control induced by HAART.

Regarding the second objective (description of the long-term CD4+ counts), we found a plateau in CD4+ increase emerging from year 4 after HAART initiation. This evidence confirms previous results showing that the increase in CD4+T-cell count after HAART had a steep initial phase, followed by a second phase during which the increase was slower but continuing in patients who maintained HIV-RNA <1,000 copies/ml [22,33-35], or reached a plateau after a variable number of years [36-38]. Furthermore, considering the individual pattern, the CD4+T-cell slopes started to decrease in several cases even before a plateau in the overall population was reached. In fact, linear regression analysis demonstrated that several patients presented a decreasing pattern from a median of approximately one year after initiation of treatment.

Decreasing CD4+ patterns could be due either to the exhaustion of the regenerative capacity of the immune system (favored by persistent immune activation despite virological suppression in the peripheral blood compartment), or to the fact that <500 HIV-RNA copies/ml as a cut-off could have covered incomplete viral suppression. While we await a demonstration that low-level viral replication, assessed by ultrasensitive assays, affects the regenerative capacity of the immune system, strict monitoring of CD4-count must be recommended even in those with long-term virological control. Moreover, we should experiment with interventions to slow or reverse unfavorable CD4+ trends and we should address whether eventual CD4+ decrease impacts on the virological and clinical outcomes of therapy.

From a clinical point of view, our results may provoke some considerations. First, we confirmed, using a large cohort over a long follow-up, that switching form PI-regimens to regimens not containing PI is a viable strategy since immune-reconstitution is not compromised. Importantly, although we did not assess clinical outcomes or quality of life, CD4+ increase has been correlated with improvement in both measures [39].

Regarding the type of regimen to which patients were switched, long-term evolution of CD4+ count did not appear to be impaired by the use of NRTI-only combinations that, owing to their suboptimal virological efficacy, have been progressively abandoned even for simplification as initial treatment of previously naive patients [23]. By contrast, in treatment simplification, the risk of virological failure to NRTI is mostly restricted to patients who have previously failed [17,18]. Moreover, all the other classes of antiretroviral drugs remain available should NRTI regimens eventually fail and HIV resistance to this class emerges. Regarding toxicity, recognized adverse events of some NRTI (e.g., anaemia due to zidovudine, or lipoatrophy due to dideoxynucleosides) were a further incentive to abandon these regimens. However, alternative regimens may provoke adverse events as well (e.g., neuro-psychological effects due to efavirenz). Furthermore, NRTI-only regimens are cheaper than the others: for instance, in Italy, the cost of treating 100 patients with co-formulated NRTI for 1 year is 998,796 Euro in comparison to 1,441,800 Euro for co-formulated NRTI+NNRTI, with a saving in direct costs of 443,004 per year). In view of these considerations, we suggest that NRTI-regimens should be re-considered for use in treatment simplification strategies, especially in resource-limited settings where other recommended options are not readily available. Obviously, this conclusion is based merely on the consideration of CD4+ count in presence of sustained undetectable HIV RNA as it was the focus in this retrospective analysis. Other important aspects were not assessed, such as adverse events, quality of life, the risk of virological failure after a switch and subsequent evolution of CD4+ count.

This work has limitations that need to be considered. The first limitation is that it was not a randomized clinical trial in which patients were randomized to continue PI or switch to alternative regimens. For example, it is possible that patients whose CD4+ counts did not increase as expected were either not switched or switched to regimens containing NNRTI but not to NRTI-only. However, we limited this bias by excluding from the study patients who continued on PI since their characteristics were too unbalanced compared with the other two groups, especially for CD4+ trend during the follow-up. Notwithstanding the exclusion of this group, a selection bias could have occurred favoring, in particular, the NRTI group. For this reason, we conducted several analyses to overcome the unbalance between the groups, even adjusting for propensity score and CD4+ count preceding the switch, all pointing to the fact that the switch and the choice of one regimen or the other did not impair the ongoing immune-reconstitution in our patients. The second limitation is that, by definition, all patients with virological failure were excluded from the study. Should patients in the NRTI group had failed more frequently, they would have been favored in this analysis. The third limitation is that, as discussed above, we used the 500 copies/ml cut-off as indicative of undetectable HIV RNA and this could have masked immune damage exerted by low-level viral load. However, ultrasensitive tests were not available for most patients at the time of the study and there is still a lack of data indicating that immune-reconstitution is different among patients with lower levels of plasma HIV load. The fourth limitation is that we did not focus on specific drugs, since we were interested in treatment strategy and consideration of multiple drugs would have reduced the power of the study to a significant extent.

## Conclusions

In conclusion, switching from first-line PI-containing regimens to NNRTI or NRTI-based regimens in the presence of <500 copies/ml HIV RNA did not appear to damage the CD4+ trend that was ongoing before the switch. Moreover, as far as long-term immunological outcome is concerned, we suggest that NRTI-only regimens be further evaluated for use in selected patients who are responding (and did not fail before) to other regimens. Lastly, we tried to provide unbiased results notwithstanding the observational nature of the study by analyzing the data from several aspects using pertinent statistical approaches. However, it is still possible that our results reflect a best case scenario regarding CD4+ immune recovery, especially in patients switched to NRTI-only. Since alternative drugs and classes are available and, for some of them, a beneficial effect on CD4+ reconstitution has been hypothesized (e.g. CCR5 co-receptor inhibitors [40]), our approach could be applied in observational studies on novel strategies using these compounds. However, possible biases intrinsic in such observational analyses are difficult to avoid. Therefore, randomized controlled trials remain the best way to study this aspect.

## Competing interests

CT and GC have received unrestricted educational grants (as speakers of for participation to conferences) from Abbott, Gilead, Merck, GSK, BMS, Schering Plough, Roche s.p.a. CM has received fees and funding from Abbott, Gilead, Merck, GSK, BMS, Pfizer, Roche s.p.a. AC has served as a consultant and received honoraria from Abbott, Gilead, Merck, GSK, BMS, Tibotec. The other authors have no financial interests to disclose in relationship to this paper.

## Authors' contributions

CT conceived of the study and drafted the manuscript; ADM provided data and revised the manuscript critically for important intellectual content; AP provided data and revised the manuscript critically for important intellectual content; GL set up the database and performed descriptive statistical analyses; GCo provided data; AA provided data; ADL provided data; CM provided data; ACa provided data; PC provided data; LM provided data; AC provided data; GC participated in study design and coordinated the study; HL participated in the statistical analysis; BMC participated in study design, performed statistical analyses and drafted the manuscript. All authors read and approved the final manuscript.

## Pre-publication history

The pre-publication history for this paper can be accessed here:

http://www.biomedcentral.com/1471-2334/11/23/prepub
